# Exosomes Enhance Adhesion and Osteogenic Differentiation of Initial Bone Marrow Stem Cells on Titanium Surfaces

**DOI:** 10.3389/fcell.2020.583234

**Published:** 2020-11-05

**Authors:** Yanhua Lan, Qianrui Jin, Huizhi Xie, Chengxi Yan, Yi Ye, Xiaomin Zhao, Zhuo Chen, Zhijian Xie

**Affiliations:** Key Laboratory of Oral Biomedical Research of Zhejiang Province, The Affiliated Hospital of Stomatology, School of Stomatology, Zhejiang University School of Medicine, Hangzhou, China

**Keywords:** osseointegration, cell–material interaction, exosomes, titanium, bone marrow stem cells

## Abstract

Successful osseointegration involves the biological behavior of bone marrow stem cells (BMSCs) on an implant surface; however, the role of BMSC-derived extracellular vesicles (EVs)/exosomes in osseointegration is little known. This study aimed to: (i) explore the interaction force between exosomes (Exo) and cells on a titanium surface; (ii) discuss whether the morphology and biological behavior of BMSCs are affected by exosomes; and (iii) preliminarily investigate the mechanism by which exosomes regulate cells on Ti surface. Exosomes secreted by rat BMSCs were collected by ultracentrifugation and analyzed using transmission electron microscopy and nanoparticle tracking analysis. Confocal fluorescence microscopy, scanning electron microscopy, Cell Counting Kit-8 (CCK-8), quantitative real-time polymerase chain reaction techniques, and alkaline phosphatase bioactivity, Alizarin Red staining, and quantification were used to investigate the exosomes that adhere to the Ti plates under different treatments as well as the morphological change, adhesion, spread, and differentiation of BMSCs. We found that exosomes were efficiently internalized and could regulate cell morphology and promoted the adhesion, spreading, and osteogenic differentiation of BMSCs. These were achieved partly by activating the RhoA/ROCK signaling pathway. Our discovery presents a new insight into the positive regulatory effect of exosomes on the biological behaviors of BMSCs on Ti surface and provides a novel route to modify the surface of a Ti implant.

## Introduction

Dental implants have become an alternative for replacing missing teeth, which are essential for patients looking to restore dental esthetics and function. Fast and effective osseointegration is central to the success of dental implant therapy (Cervino et al., 2019). Successful osseointegration benefits from improved surface modification methods. Studies related to the implant surface have increased exponentially, largely focusing on two aspects: abiotic methods (chemical and physical methods) based on surface engineering (Kuroda et al., 2002; Omar et al., 2016) and biotic methods (cells, polypeptide, covalent grafting, and etc.) that alter the implant surface with functional biomaterials (Zhang et al., 2011; Liu et al., 2017). The advantages of biotic implant modification, such as high osteoinduction capability, biocompatibility, and biodegradability, are required in many cases for biomedical applications (Geetha et al., 2008). However, the use of these traditional biotic implant modifications has several disadvantages, such as explosive release, immunogenicity, instability, and easy degradation. Recently, it has been proven that nanoscale materials that show novel physical, chemical, and biological properties with high stability could be efficient alternatives to increased cell responses, bone conduction, and osseointegration (Wang Y. et al., 2018).

Osseointegration and the maintenance of bone homeostasis on the bone–implant contact (BIC) mostly rely on cell-to-cell and cell-to-material communication, and this osseointegration process is directed by complex biological mechanisms that involve many cells and their secreted signaling factors (Nagasawa et al., 2016). Bone marrow stem cells (BMSCs) play an essential role in some key processes of osseointegration involving osteoinduction, osteogenesis, and bone reparative mechanism (Won et al., 2017). *In vitro* and *in vivo* studies suggest that various “tiny pieces of matter” (secreted by cells) such as cytokines, chemokines, growth factors, and others are implicated in the regulation of BMSC biological behavior. However, little is known about events in the interaction and regulation of cell-derived secretome products and the biological behavior of BMSCs.

Exosomes (Exo), specifically defined as the 50- to 200-nm vesicles that are secreted by multiple cells, have been reported to be present in biological fluids and are involved in multiple physiological and pathological processes. Exosomes are now considered an additional mechanism for intercellular communication, allowing cells to exchange proteins, lipids, and genetic material (van Niel et al., 2018). Among the multifarious exosomes, mesenchymal stem cell exosomes (MSC-exosomes) have attracted great attention as they have recently been identified as possibly functioning as regulators of various treatments, especially tissue engineering, and tissue regeneration.

Mesenchymal stem cell-exosomes, like most exosomes that carry informative cargo from the MSC to targeted cells, influence fundamental cellular processes including apoptosis, proliferation, migration, and lineage-specific differentiation (Brennan et al., 2020). Within the field of orthopedics and dentistry, MSC-exosomes regulate the osteogenic differentiation of MSCs by transferring vital materials, such as osteogenesis-related protein and microRNAs (Wang X. et al., 2018). Moreover, many studies have shown that multiple regulatory factors and complex signaling pathways involved in the process of osteogenesis differentiation are regulated by MSC-exosomes. Specific pathways including Wnt, BMP, PI3K/Akt, insulin, TGFβ, and calcium signaling pathways may be affected by MSC-exosomes (Cooper et al., 2019; Wei et al., 2019; Zhang et al., 2020). In aggregate, these researches demonstrate that MSC-exosomes carry much information that impacts key gene activation for osteogenesis including SATB2, Runx2, Dlx5, and Osterix (Osx; Fang et al., 2015; Huang et al., 2017). Despite extensive research, a clear picture is yet to emerge on how MSC-exosomes regulate cell biological behavior and differentiation, especially in materials frequently used for implant application.

Exosomes are certainly nanoscale intercellular messengers secreted by cells to deliver biological signals. Thus, the “if and how” they regulate the behavior of BMSCs on titanium (Ti) or other materials have become interesting and intriguing (Al-Sowayan et al., 2020). Furthermore, considering the outstanding properties of exosomes (natural origin, cargo representing a rich source of factors, and low immunogenicity), there may be a novel strategy to promote the activity of BMSCs in the process of osseointegration by introducing exosomes.

Therefore, the purposes of this study were to: (i) explore the form of the interaction force between exosomes and cells in a Ti environment; (ii) discuss whether the morphology and biological behavior of BMSCs are affected by exosomes; and (iii) preliminarily trace the internal molecular mechanism of this regulation on a Ti surface.

## Materials and Methods

### Treatments With Titanium

**FIGURE 1:**
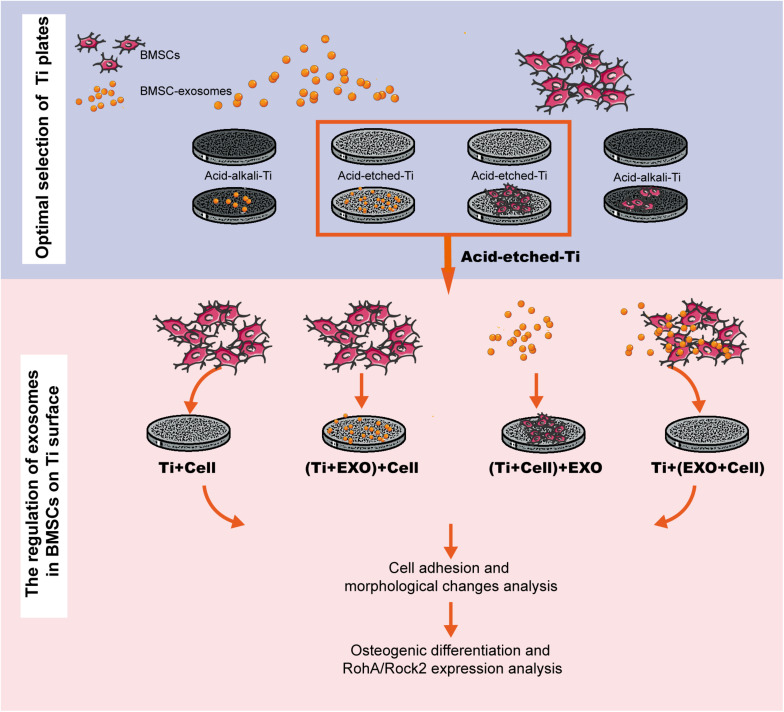
Experimental flowchart. This workflow is divided into two steps: selecting a favorable adsorption material for exosomes and cells and investigating the regulation of exosomes in bone marrow stem cells (BMSCs) on Ti surfaces in three different combinations. BMSC-derived exosomes (*Exo*).

Pure Ti plates (grade 4, 10 × 10 mm, 1-mm thickness; Guangci Medical Appliance Company, Zhejiang, China) were polished by grinding using silicon carbide (*SiC*) abrasive paper (#320 to #1600 grit sizes). Acid-etched Ti plates were treated as described in Zhang et al. (2011). Pure Ti plates were sand-blasted, rinsed with acetone, ultrasonic cleaned in 75% alcohol and distilled water, and dried under nitrogen flow. Ti plates were then treated with a solution containing hydrofluoric acid (HF) and HNO_3_ followed by treatment with a solution containing HCl and H_2_SO_4_. On the other hand, the acid–alkali Ti plates were treated as follows (Jin et al., 2017). The Ti plates were treated with a solution containing HF and HNO_3_, ultrasonically cleaned in 75% alcohol and distilled water, and treated with a solution containing 5 M NaOH for 30 min. Then, the Ti plates were heated to 450^°^C, kept for 1 h, and cooled to room temperature (25^°^C) naturally. All Ti plates were sterilized by ultraviolet (UV) radiation before use in the experiment. The experiment exclusively investigated the adsorption and biological effects of exosomes on BMSCs cultured on a Ti surface (Figure 1).

### rBMSC Extraction, Culture, and Conditioned Medium Collection

Rats were sacrificed by an overdose of chloral hydrate to obtain rat bone marrow stem cells (rBMSCs) for culture. These cultures were prepared according to the protocol developed in Caplan Laboratory and previously carried out in our laboratory (Ke et al., 2016). The experimental procedures were approved by the Ethics Committee for Animal Research at Zhejiang University (ethics approval number: ZJU20200075). In brief, the tibia and femur, without attached tissues, were excised under sterile conditions. Subsequently, bone marrow was extracted using an injection of basal growth medium (BGM) consisting of α-MEM medium (HyClone, UT, United States) with 10% fetal bovine serum (FBS; Gibco, New York, United States), 100 U/ml penicillin, and 100 μg/ml streptomycin. rBMSCs from passages 3–5 were used in this experiment. When cells were needed for differentiation into osteocytes, the rBMSCs were cultured in an osteogenic differentiation medium supplemented with 10 mM β-glycerophosphate, 50 μg/ml ascorbic acid, and 10^–8^ M dexamethasone (Sigma-Aldrich Co., St. Louis, United States). Besides, adenoviral vectors encoding a green fluorescent protein (Ad-GFP; GeneChem Co. Ltd., Shanghai, China) were used at a multiplicity of transduction of 100 for a clear presentation of the morphology of BMSCs.

### Characterization of r-BMSCs by Flow Cytometry

Attached cells were trypsinized and detached from the polystyrene flask and separately incubated with the phycoerythrin-conjugated anti-rat antibodies, anti-CD29, anti-CD90, anti-CD45, and anti-CD34 (Biolegend, United States), for 30 min at room temperature. Then, the cells were washed twice with phosphate-buffered saline (PBS), centrifuged (1,500 rpm, 5 min) at room temperature, and resuspended in PBS. The cells were adjusted to a concentration of 5 × 10^5^ cells/100 μl. Flow cytometric analysis was carried out using FACScan system (BD Becton Dickinson, United States).

### Isolation of Exosomes

**FIGURE 2:**
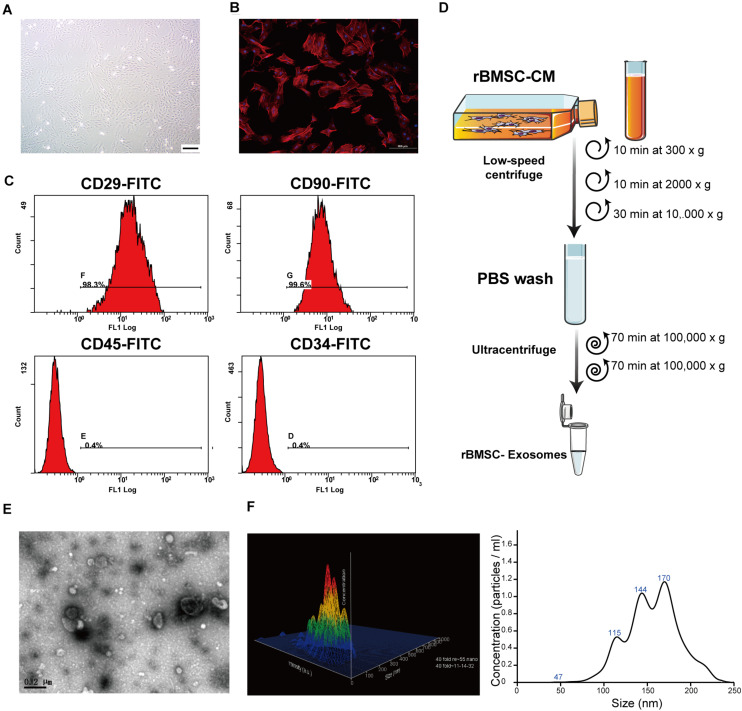
Rat bone marrow stem cell (rBMSC) and exosome identification. **(A)** Typical photograph of spindle-shaped rBMSCs (undifferentiated) under an inverted microscope (*scale bar*, 50 μm). **(B)** Identification of rBMSC morphology through confocal microscopy (*scale bar*, 200 μm). **(C)** Flow cytometric analysis to identify rat BMSCs. The presence of rBMSCs was confirmed by positive staining for CD29 and CD90 as well as negative staining for CD34 and CD45. *CD*, cluster of differentiation. **(D)** Exosome purification protocol: collect conditioned medium from rBMSCs, use low-speed centrifugation and filtration to remove dead cells and debris, and wash contaminating proteins using an ultracentrifuge. **(E)** Representative image of exosome morphology as captured by transmission electron microscopy (TEM; *scale bar*, 200 nm). **(F)** Measurement of the particle size distribution of exosomes by nanoparticle transport analysis (NTA).

Before exosomes were extracted from rBMSCs, a fresh serum-free medium was introduced after cell attachment. A cell-conditioned medium was obtained from the rBMSCs cultured for 2 days in a medium without added serum and subjected to differential centrifugation (300 × *g*, 10 min; 2,000 × *g*, 10 min; and 10,000 × *g*, 30 min) at 4^°^C and filtered through a 0.45-μm membrane to remove large debris and dead cells. Then, the exosomes were pelleted by ultracentrifugation at 100,000 × *g* for 140 min at 4^°^C using a 70 Ti rotor (Beckman Coulter, Fullerton, United States; Théry et al., 2006). Finally, the supernatant was removed and the pellet resuspended in cold PBS throughout the ultracentrifugation step trials. A schematic of the exosome sample preparation method is shown (Figure 2D).

### Characterization of Exosomes

After collecting the isolated exosomes, their morphology was observed using transmission electron microscopy (TEM). In brief, the exosomes were fixed with 4% paraformaldehyde (PFA, 1:1; Alfa Aesar, United States), then loaded onto a TEM copper grid, and washed with PBS, 1% glutaraldehyde, and distilled water. Exosome samples were next incubated with 4% uranyl acetate and visualized under a Tecnai 12 BioTWIN TEM (FEI/Phillips, United States) at 100 kV. Additionally, the number and the size distribution of exosomes were analyzed by nanoparticle trafficking analysis (NTA) using a NanoSight NS300 system (Malvern, United Kingdom) according to the manufacturer’s instructions. In brief, the exosome samples were diluted, resuspended in 1 ml PBS, and reinjected into the device (Liao et al., 2019).

### Optimal Selection of Ti Plates

The protein concentration of exosomes was determined using a bicinchoninic acid (BCA) assay with a standard curve (Biyuntian Biotech Company, Shanghai, China). The concentration of each exosome sample was measured and adjusted to 100 μg/ml using cold PBS. One milliliter of the exosome solution was added dropwise to the surface of different Ti plates (two groups: acid-etched Ti and acid–alkali Ti). After 5 min of incubation at 4^°^C, the exosomes were immobilized on the surface of the Ti plates. The remaining solution was removed, collected, and the protein concentration determined to calculate the quantitative detection of the adsorption of exosomes by different Ti plates. rBMSCs were seeded at a low density (5,000 cells/plate) and grown overnight in order to screen and quantify exosome-regulated adhesion behavior of rBMSCs on two types of Ti surfaces.

### Inoculate BMSC and BMSC-Exosome on Ti Plates

To examine the regulatory role of exosomes on rBMSCs cultured on Ti plates, the rBMSCs and exosomes were seeded on acid-etched Ti plates in four different combinations (Figure 1): Ti + Cell group: 100 μl cell suspension (2 × 10^5^ cells/ml); (Ti + Exo) + Cell group: 100 μl of exosome suspension (100 μg/ml) was seeded after 12 h incubation at 37^°^C, 100 μl cell suspension; (Ti + Cell) + Exo group: 100 μl cell suspension was seeded 12 h before, 100 μl exosome suspension; and Ti + (Exo + Cell) group: 100 μl cell suspension and 100 μl exosome suspension were premixed before being added on Ti plates. The suspension was dropped onto the surface of Ti and allowed to settle for 15 min before the addition of a culture medium.

### Confocal Fluorescence Microscopy and Image Analysis

Purified exosomes were labeled using PKH67 green fluorescent cell linker (PKH67GL-1KT; Sigma-Aldrich Co.) according to the manufacturer’s instructions and then added to low-density rBMSC suspensions (5,000 cells/10 μg exosome) in each Ti plate. After 6 h of incubation, the Ti plates were washed three times in PBS and then fixed with 4% paraformaldehyde. The cell F-actin was visualized using rhodamine–phalloidin (Cytoskeleton Inc., Denver, United States). The nuclei were stained using DAPI (Sigma-Aldrich) following the manufacturer’s recommended protocol.

### Observation of Adherent Cells on Ti Plates Using Scanning Electron Microscopy

The adhesion of rBMSCs and the physical location and interactions between the cells and exosomes on the Ti surface were evaluated using a field-emission scanning electron microscope (FE-SEM; SU8010, Hitachi, Tokyo, Japan). In summary, the acid-etched Ti plates loaded with cells and exosome samples were fixed in 2% paraformaldehyde at 4^°^C for 15 min, washed with 0.15 M sodium cocoate buffer, and stained with 1% osmium tetraoxide for 2 h. After rinsing with the buffer, the samples were dehydrated through an ethanol series (30 min each in 50, 60, 70, 80, 90, 95, 100%, and dry ethanol) and dried in the air before gold sputter coating and observation (Wang Y. et al., 2018).

### ALP Bioactivity

Alkaline phosphatase (ALP) bioactivity was examined by ALP staining and ALP activity testing after 7 days. For ALP staining, the acid-etched Ti plates loaded with cells and exosome samples were fixed in 4% paraformaldehyde and stained using a BCIP/NBT ALP color development kit (Biyuntian, China), then washed with PBS and photographed. ALP activity was determined using the LabAssay ALP kit (Wako Pure Chemical Industries, Japan) according to the manufacturer’s instructions. Total cellular proteins were determined using the BCA protein assay and the absorbance at 405 nm was measured with a Microplate reader (Thermo Fisher Scientific).

### Alizarin Red Staining and Quantification

Alizarin Red S stain was used for staining mineralized nodules. Briefly, the Ti plates loaded with cells and exosome samples were fixed in 4% paraformaldehyde and stained by 2% Alizarin Red S for 30 min. For quantification of Alizarin Red S staining, the samples were fixed in 70% ethanol for 1 h, then washed three times with dH_2_O and stained with 0.5% Alizarin Red S solution (*w*/*v*, pH 4.2). The stain was desorbed with 10% cetylpyridinium chloride for 30 min and the absorbance at 562 nm was measured.

### Cell Adhesion and Spread

Two methods were used to investigate the initial adhesion behavior of rBMSCs on the Ti plates. One method involved counting the attached cell numbers by counting the number of DAPI-stained nuclei in five random fields, expressed as the average number of the positively labeled cells per unit area of view. Then, the numbers of adherent cells in the co-culture setting with/without exosomes were indirectly quantified using the Cell Counting Kit-8 (CCK-8; Dojindo Laboratories, Kumamoto, Japan). Next, the spreading area of the cells was calculated using ImageJ analysis software. The average cell spreading area was calculated as *S* = Subtotal/Number of Nuclei, where Subtotal is the total cell spreading area on the image.

### Quantitative Real-Time Polymerase Chain Reaction

The expression levels of cell adhesion, spreading, and osteogenic genes were evaluated using quantitative real-time polymerase chain reaction (qRT-PCR) for marker genes including the ras homolog family member A (RhoA), Rho-associated coiled-coil containing protein kinase 2 (ROCK2), Osterix (Osx), osteocalcin (OCN), and ALP in rBMSCs. Total RNA was isolated and purified using an RNeasy kit (Qiagen GmbH, Hilden, Germany). Complementary DNAs (cDNAs) were synthesized using a PrimeScript RT Master Mix (Takara Bio, Osaka, Japan). qPCR analysis was performed in duplicate for each sample in a 10-μl reaction using a CFX384 real-time system (Bio-Rad Laboratories, California, United States) with an SYBR Green I kit (Takara City, Osaka, Japan). All the expression levels of the target gene were normalized to those of the housekeeping gene, GAPDH. The primer sequences used are listed in Supplementary Table 1.

### Statistics

All tests were repeated three times with two parallel runs for each assay, for a total of six runs, and the results presented as means ± standard deviation. Independent *t* test statistics were computed using SPSS version 19.0 (SPSS, Chicago, United States) and the *p* value set at 0.05.

## Results

### Characterization of rBMSC and Exosomes

To comprehensively characterize the purified nanoparticles derived from rBMSCs, TEM, and NTA were employed. All exosomes are derived from donor cells (rBMSCs) in good condition, which appeared spindle-like in shape on the tissue culture plate (Figures 2A,B). The cell surface markers were determined by flow cytometry analysis to identify rBMSCs. The rBMSCs were confirmed by high percentages of positive staining for CD29 (98.3%) and CD90 (99.6%) as well as by low percentages of staining for CD34 (0.4%) and CD45 (0.4%; Figure 2C). The morphology of the BMSC-derived exosomes was first confirmed in the TEM image (Figure 2E), which revealed that the exosomes were 100- to 200-nm spherical particles with a complete membrane structure and similar shape to a biconcave-discoid, fitting the recognized characteristics of exosomes. The size distribution of the nanoparticles in exosomes is shown in a relative-intensity three-dimensional plot, which is characterized by NTA (Figure 2F). The concentration of exosomes was 3 × 10^8^ particles/ml and the mean diameter of the particles was 163.7 ± 1.6 nm, as measured by the NTA system. Taken together, these data suggest that the nanoparticles are exosomes.

### The Different Morphologies and Adsorption Capacity of Titanium Surface Under Acid or Acid–Alkali Treatments

**FIGURE 3:**
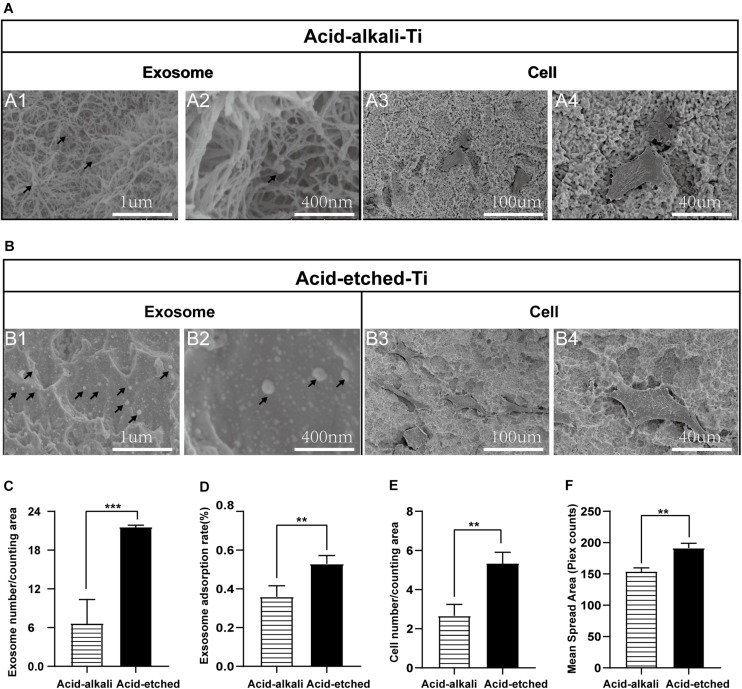
Rat bone marrow stem cell (rBMSC) and BMSC-exosomes adhesion rates on the acid-etched Ti and acid–alkali Ti plates. **(A)** SEM images: Exosomes and cells on acid–alkali Ti surfaces (*scale bar*: *A1*, 1 μm; *A2*, 400 nm; *A3*, 100 μm; and A4, 40 μm). **(B)** SEM images: Exosomes and cells on acid-etched Ti surfaces (*scale bar*: *B1* 1 μm; *B2*, 400 nm; *B3*, 100 μm; and *B4*, 40 μm). **(C)** The number of exosomes was counted under SEM. **(D)** The adherence intensities of exosomes were quantified. The protein concentration indicated the quantity of exosomes adhering to the Ti surface. **(E)** The number of rBMSC cells was counted under SEM. **(F)** The average steady-state spreading area of each rBMSC was quantified using ImageJ. The results are presented as the mean ± standard error of the mean, *n* = 3. *p < 0.05; **p <‘0.01, ***p < 0.001. The *black arrow* indicates exosomes adhering to the Ti surface.

For qualitative and quantitative evaluation, the adsorption capacities of two Ti surfaces (acid-etched and acid–alkali) were evaluated by SEM and protein quantification analysis of the leaching solution. Ti plates were sandblasted and acid-etched to obtain an irregular and rough surface with macro- and micro-pits similar to those commonly used in commercial dental implants (Figure 3A, 1–4). SEM images of the nanostructures were prepared using Ti wet corrosion in 5 M NaOH aqueous solution at 450^°^C. Porous nanowire networks were formed on the acid–alkali Ti surface. The nanowire networks were entangled, featuring a coralline-like morphology at a low magnification with a multilayered overlapping stamen shape at a high magnification (Figure 3A). To our surprise, such a complex morphology was unfavorable to the adherence of exosomes and cells when comparing with acid-etched surface (Figures 3A,B). It is clear from the pictures that the number of adherent exosomes on the acid-etched Ti surface was about two times greater than that on the acid–alkali Ti surface (Figure 3D). The acid-etched Ti surface has advantages over the numbers of cells and exosomes per counting area (Figures 3C,E) as well as the mean spreading area of the cells. Therefore, the acid-etched Ti plates were selected for subsequent experiments due to this greater ability to adsorb exosomes and cells. To sum up, acid-etched Ti has a stronger adsorption of exosomes and is therefore more likely to lead to the effective realization of the potential of exosomes.

### Exosomes Induce Morphological Changes in BMSCs

**FIGURE 4:**
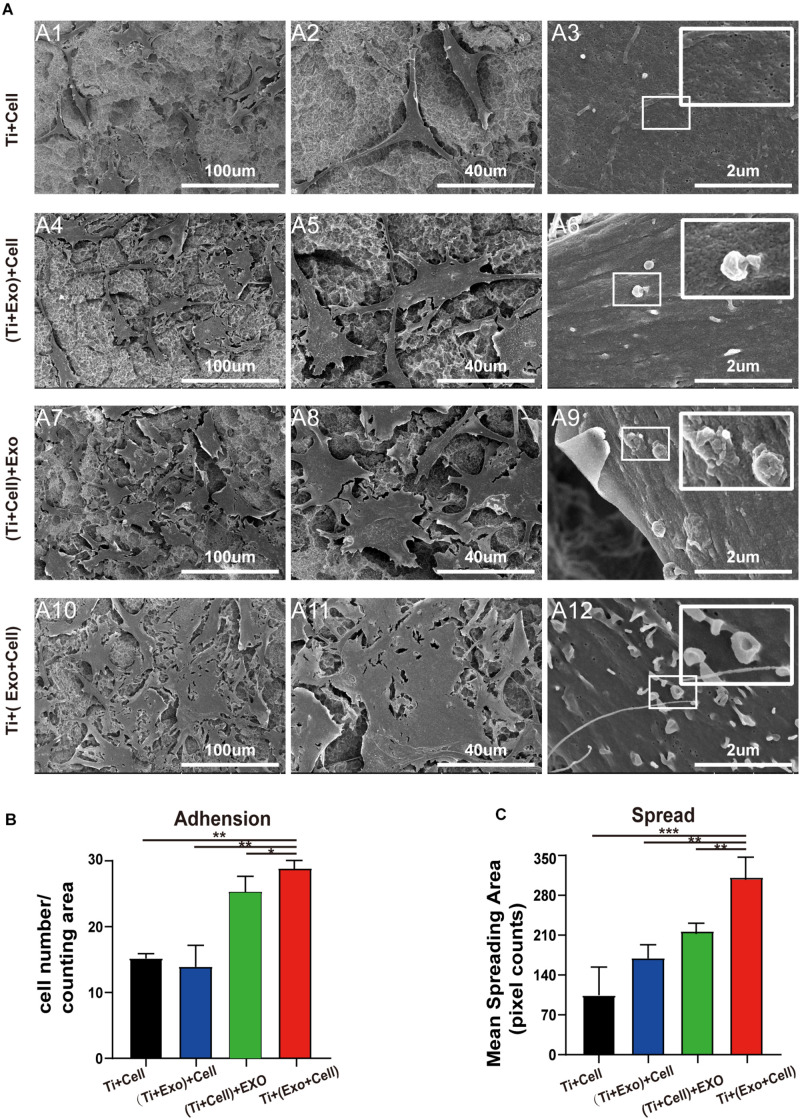
SEM images of adherent rat bone marrow stem cells (rBMSCs) under different treatments of exosomes. **(A)** No exosomes appear on the control surface and adherent cells. In the Ti + (Exo + Cell) group, rBMSCs with longer and more numerous microvilli are detected in the presence of exosomes compared with the other three groups. The high-magnification image shows the endocytosis of exosomes by BMSCs (*scale bar*: *A1*, *A4*, *A7*, and *A10*, 100 μm; *A2*, *A5*, *A8*, and *A11*, 40 μm; *A3*, *A6*, *A9*, and *A12*, 2 μm). **(B)** The number of attached cells was counted under SEM. **(C)** The average steady-state spreading area of each rBMSC was quantified using ImageJ. The results are presented as the mean ± standard error of the mean, *n* = 3. *p < 0.05; **p < 0.01, ***p < 0.001. *ns*, not significant; *Exo*, exosomes.

To understand whether the physical interactions occurred preferentially between exosomes and cells, we next carefully observed them when both were present on the Ti surface. Based on our experimental results listed above and pilot experiments, the acid-etched Ti plates were chosen for subsequent experiments. Interestingly, as we observed in the low-magnification SEM images of the Ti surface, the exosomes were more likely to adhere to the surface of the cells while it was hard to see exosomes that adhered to the Ti surface away from the cell body. Moreover, changes in the cell morphology induced by exosome treatment were particularly obvious in the Ti + (Exo + Cell) group, including elongated, spindle-shaped morphology, pseudopodia formation, and increased cell scattering (Figure 4A). In contrast, cells showed no/decreased microvilli or filopodia- and lamellipodia-like structures on the Ti surface without exosomes. These results indicate that exosomes are more inclined to bind and be internalized by cells on the Ti surface. In addition, the adhesion and spreading of rBMSCs on the Ti surface were stimulated with the addition of exosomes (Figures 4B,C).

### Exosomes Promote BMSC Adhesion and Spread on Ti Surfaces

**FIGURE 5:**
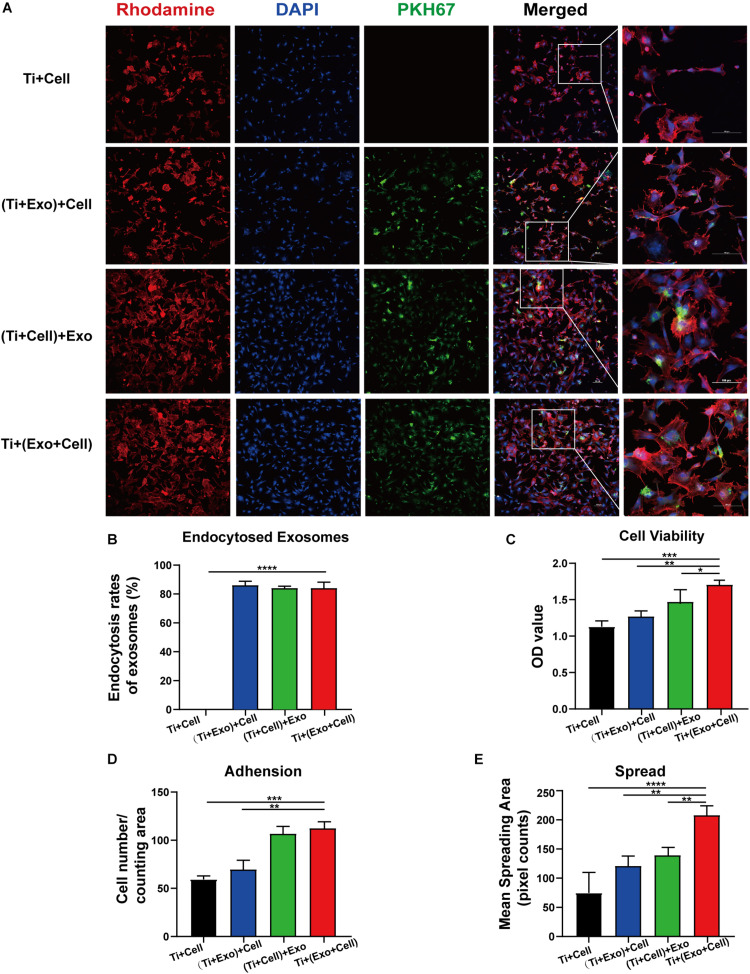
Exosomes specifically bind to rat bone marrow stem cells (rBMSCs) and promote the adherence of rBMSCs onto Ti surfaces. The cells with or without exosome treatment were imaged using a confocal microscope. **(A)** rBMSCs incubated with or without PKH67-labeled exosomes (*green*) for 12 h. The nuclei of rBMSCs were stained using DAPI (*blue*), while the actin cytoskeleton was stained using phalloidin–rhodamine (*red*). *Scale bar*, 100 μm. **(B)** *Bars* represent the exosome endocytosis rates given as (number of cells containing exosomes/number of cell in each region) × 100. **(C)** The cell viability of adherent cells was measured using a CCK-8 assay after 12 h. **(D)** The number of attached cells was counted under a microscope. **(E)** The average steady-state spreading area of each rBMSC was quantified using ImageJ. The results are presented as the mean ± standard error of the mean, *n* = 3. *p < 0.05, **p < 0.01, ***p < 0.001, ****p < 0.0001. *ns*, not significant; *Exo*, exosomes.

Having shown that exosomes are efficiently internalized by cells, we then investigated whether exosomes play a role in the adhesion and spread of rBMSCs. We first proved that an interaction occurred between the cells and exosomes by tracing exosomes after co-culturing for 12 h and immunofluorescent staining in all the experimental groups (Figure 5A). Surprisingly, approximately 80–90% of cell-endocytosed exosomes without statistical differences were found regardless of the sequence of exosome treatment (Figure 5B). Then, the cell adhesion was directly and indirectly quantified by confocal microscope counting (Figure 5D) and CCK-8 assay (Figure 5C), respectively. Similarly, cell spread was calculated using ImageJ analysis software (Figure 5E). Over the same culture time, when observed by a microscope, the number of adhered cells in the Ti + (Exo + Cell) group was significantly more than that in the no-exosome group (Figure 5A). Also, rBMSCs in the Ti + (Exo + Cell) group with exosomes showed a significantly higher optical density (OD) value, which means an increased number of live cells (Figure 5C). The consistent results of the metabolic activity measurement and quantitative morphological assessment of the live cells were observed in the number of adherent rBMSCs. A similar result was obtained: under culture conditions in the presence of exosomes, treated rBMSCs displayed a dramatically increased adhesion to Ti surfaces compared with the control (Figure 5D). The cell spread was evaluated by measuring the mean spreading area of each cell (Figure 5E). The cell spreading area was significantly greater on exosomes than on the control Ti surface. In summary, the consistent results of the metabolic activity and quantitative morphological of cells were observed in the number of adherent rBMSCs, which indicated that exosomes had obvious cell tropism and contributed to the adhesion of rBMSCs on Ti surfaces.

### The Expression of Selected Adherence and Osteogenic Differentiation Markers in rBMSCs on Ti Surfaces Were Upregulated by Exosomes

**FIGURE 6:**
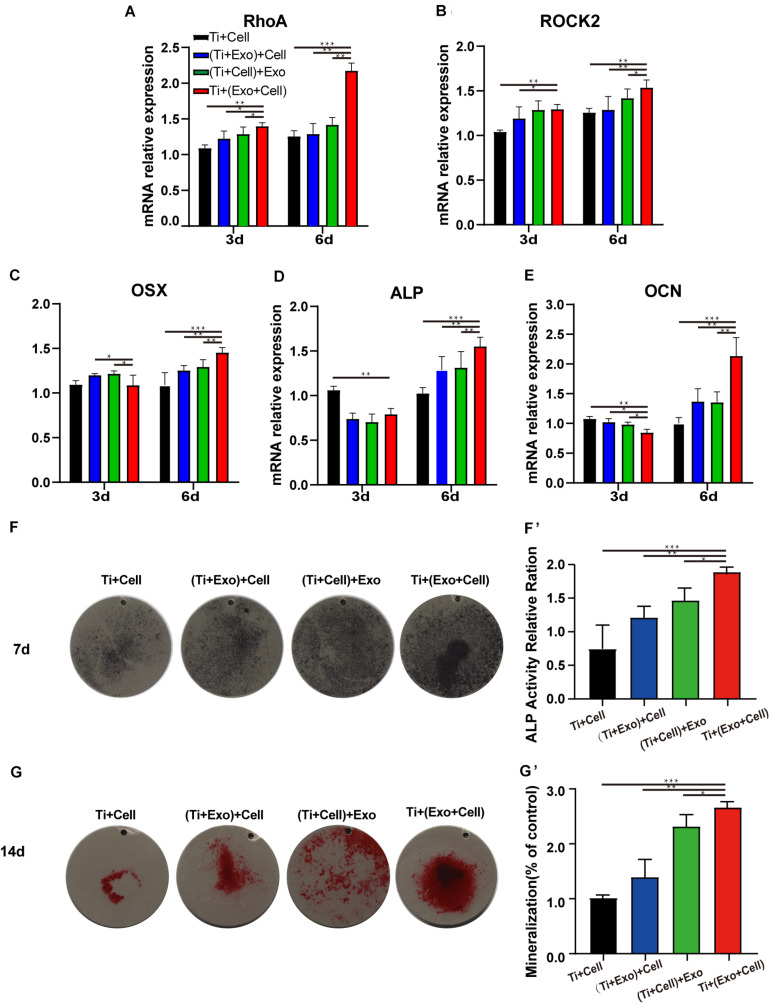
Exosomes induce elevated expressions of genes related to cell adhesion (RhoA and ROCK2), improving osteogenesis in rat bone marrow stem cells (rBMSCs). The levels of gene expression of the cell adhesion markers RhoA **(A)** and ROCK2 **(B)** and the osteogenic differentiation markers Osterix **(C)**, ALP **(D)**, and OCN **(E)** in rBMSCs on Ti surfaces at different treatments of exosomes were analyzed. **(F)** The formation of mineralized nodules was then observed by Alizarin Red S staining. **(G, G′)** The quantitative alkaline phosphatase (ALP) assay was a parallel experiment to ALP staining. **(F, F′)** Alizarin Red staining was measured and quantification is shown on the *right*. The results are presented as the mean ± standard error of the mean, *n* = 3. *p < 0.05; **p < 0.01, ***p < 0.001. *ns*, not significant; *Exo*, exosomes.

We next asked whether exosomes could contribute to the osteogenic differentiation of BMSCs and whether cytoskeletal changes were relevant. Exosomes significantly upregulated the rBMSC expression of cell adhesion, spread, and migration compared to those on the control Ti (Figures 6A,B). Especially in the Ti + (Cell + Exo) group, the effect was more pronounced. In comparison with the Ti group, the expression levels of RhoA and its downstream molecule ROCK2 were both up to nearly 2 and 1.5 times higher, respectively.

Osteogenesis-related genes’ (Osx, OCN, and ALP) mRNA expressions in rBMSCs co-cultured with exosomes were determined by real-time PCR (Figures 6C–E). At 6 days, all selected osteogenic genes were upregulated on the Ti with exosomes. Increases of Osx, OCN, and ALP ranging from 1.5 to 2 times in the Ti + (Cell + Exo) group were observed when compared with the other three groups. To summarize, exosome treatment promoted the upregulation of RhoA and ROCK2, which indicates activation of the RhoA–ROCK pathway. Moreover, exosomes promoted significant ALP, Osx, and OCN temporal upregulation by day 6 in the osteogenic differentiation medium, especially when they were premixed with rBMSCs in the Ti + (Cell + Exo) group.

Bone mineralization resulting from calcium deposition is known as a late marker in osteogenic differentiation. The results of the Alizarin Red and ALP staining and quantitation (Figures 6F,G′) illustrated that there were more calcified nodules and higher ALP staining intensity when co-cultured with exosomes. These effects were most apparent in the Ti + (Cell + Exo) group. The ALP and Alizarin Red quantitations were elevated around 1.8- and 2.2-fold, respectively.

## Discussion

Effective integration of dental implants requires the chronological processes of osteoinduction, osteoconduction, and osseointegration (Albrektsson and Johansson, 2001). Cell-to-cell and cell-to-material communications are indispensable to these osseointegration processes, especially in the early stages (Smeets et al., 2016). Of interest in this study, we investigated the exosome trafficking between cells that take part in the regulation of the biological behaviors of BMSCs on the Ti surface. Here, cell adherence, spread, and osteogenic differentiation, which are crucial biological behaviors in the process of osseointegration, were evaluated. The main finding of the present work was that exosomes efficiently internalized and modulated cell biological behaviors on the Ti surface, most likely by activating the RhoA–ROCK pathway.

Firstly, we observed that exosomes have preferences for a special landing position. Our results suggest that the affinity of exosomes was strongest for cells, weaker with an abiotic surface, and quite reduced on an acid–alkali Ti surface compared to acid-etched Ti. The authors concluded that better adhesion might result from the physicochemical properties of materials and, more specifically, the different affinity with water and surface charge. One possible reason is that the acid–alkali Ti surface usually shows a lower water contact angle, which means higher surface energy, better wettability, and, thus, stronger hydrophilicity (Hoshino et al., 2015). However, exosomes have an intrinsic favorable lipid and surface protein composition, leading to an easier adherence to hydrophobic followed by super-hydrophilic substrates. Another possible reason is that the exosomes which demonstrated negative zeta potential values of about −10 to −50 mV (Maroto et al., 2017) tend to be more attracted to the positively charged surface and might be more prone to trapping on the acid-etched Ti surface (with a large number of protons) easily. Experiments show that the acid–alkali Ti, which is widely accepted and clinically used, has better performance in exosome adhesion. The results of the study support the use of acid–alkali Ti as a good system to study exosome-based modification of Ti surfaces.

In our following study, we found that exosomes and cells produced a very interesting attractive interaction when there were coexisting BMSC–exosomes in the cell milieu. We observed that the sequence of addition of exosomes and cells led to a different cell behavior promoted by exosomes. Compared to the other two sequences, the premix maximizes the effect of adhesion and spreading, wherein the full contact and interaction between the exosomes and cells are feasible. More interestingly, a common point in these observations is that this interaction appears to be cell tropism. Exosomes present where the cells reside, but are not sufficient to affect the positions the cells adhered to. However, Wang et al. (2020) found that exosome-triggered effects mainly occur when the exosomes are immobilized on the surface, but do not occur when exosomes are provided in suspension. This may be partially explained by the direct exposure of the assembled exosomes on the smooth surface of titanium. In contrast to the previous work, titanium with an acid-etched surface and three-dimensional structure was examined in the present study, which is more commonly used in clinical practice. These exosomes likely stuck to cells to mediate the cell adhesion and migration to the Ti surface. The spatial location relation between the exosomes and cells was determined for the cellular uptake of exosomes (Figures 4, 5). This phenomenon that exosomes are easily internalized by cells was first identified in cancer studies and is called “efficient cellular internalization.” This same feature (with four other features: long circulation, enhanced tumor accumulation, deep tumor penetration, and drug release) makes exosomes a novel tool in non-invasive anticancer therapy (Yong et al., 2020). However, this phenomenon exists not only in the tumor microenvironment but also on the Ti surface. Although the mechanisms of the processes remain unclear, they might shed light on previous studies in the field of tumor biology. The proposed mechanism of enhanced efficient cellular internalization can be attributed to the following three aspects: (i) as Smyth et al. (2014) have shown, in addition to the lipid component, the unique protein composition of exosomes promotes their internalization in cancer cells. Evidence indicated that ligand–receptor binding played an important role in promoting cellular internalization (van Dongen et al., 2016). (ii) There are some targeting ligands expressed on exosomes that are responsible for efficient cellular internalization. For example, it has been demonstrated that glycans and the soluble ligand CCL18 on extracellular vesicles (EVs) derived from cells mediated the interaction between exosomes and cancer cells (Islam et al., 2019). (iii) Extracellular matrix remodeling (ECM) is another cytosolic protein that plays an important role in efficient cellular internalization. Hoshino et al. have pointed out that the unique exosome integrin interaction with the cell-related ECM could mediate exosome uptake in specific target organ cells. In this case, ECM can be treated as a “zipper” between the exosomes and integrin on target cells (Hoshino et al., 2015). We set out to understand the efficient cellular internalization of exosomes. Exosomes also play an important role at the interface between the implant and bone tissue. The finding implies that exosomes may have advantages of targeting and specificity as bioactive-based materials (Zhou et al., 2020).

Of great importance is that exosomes are not only internalized by cells but also possess a robust capacity to affect the cell morphology and cell adhesion on the Ti surface (Wei et al., 2019). The number of mast cells was significantly higher on the Ti surface with the treatment of exosomes. Adherent rBMSCs on the Ti surface with exosomes appeared more stretched and larger than those on the control Ti surfaces. Concurrently, significant morphological characteristics (more triangular or polygonal and branched and filopodia-rich) of the rBMSCs were observed. Cell morphology contributes as a descriptor, indicator, or intermediate factor in characterizing cell material interactions and reflects the integrative effect of many distinct processes and signaling pathways across different scales, and it may be a valuable descriptor of cell behaviors in differentiation and function (Marklein et al., 2016; Islam et al., 2019). For example, the study of Marklein et al. (2016) found that cell morphology could also serve as a predictor of the fate of progenitor cells. This beneficial effect is promoted by the exosome-mediated factor communication, which led to cell cytoskeleton rearrangement, adhesion, spread, and differentiation. However, the precise mechanisms for the enhanced exosome-related MSC spreading and adhesion are not clear yet. One possibility is the exosome-mediated transfer of molecular signaling among cells. A candidate may be RhoA, a molecule facilitating cytoskeleton remodeling and formation of adherens junctions. Through its effect on ROCK-mediated cytoskeletal tension, RhoA directly mediates the shape-related control of lineage stereotypes. As McBeath et al. (2004) said, controlling RhoA activity could even replace the need for soluble differentiation factors that caused osteogenesis. We did find that the expressions of RhoA and ROCK2 significantly increased under the treatment of exosomes, which is in line with our hypothesis. Furthermore, as suggested by previous studies, the phenomenon can be further explained by the transfer of an important signaling molecule. Wang et al. (2020) using proteomic analysis, suggested that MSC-exosomes carry a robust profile of cell adhesion molecules and signaling molecules, such as integrins, cadherins, and fibronectins. In the present study, we demonstrate that exosomes can regulate cell behaviors by upgrading the activation of the RhoA–ROCK pathway, but what triggers the signaling pathway requires further exploration.

Besides its adhesion impact, compelling evidence indicates the great potential of BMSC-exosomes for bone tissue engineering. The mechanism of the effect of exosomes on the increase of BMSC spreading and adhesion is still unclear. It is reasonably believed that osteogenic differentiation is related to the development of cell density (Pena et al., 2019), especially as we have previously shown that exosomes promote BMSC adhesion and cell–cell contact. Several studies suggest that cell density, which impacts cell–cell contacts and the concentration of paracrine factors, plays a role (Kempf et al., 2014). Moreover, a positive combined effect of adhesion and proliferation was found in some previous *in vitro* studies on different biomaterial surfaces (Reher et al., 2017). As the metal implants persist for the long term, the progress of dental implant osseointegration is distinct from normal bone defect repair. Highlighted as a key in this study is the efficient cell adhesion and spreading on the Ti surface. The secretion and the reciprocal exchange of exosomes between adherent cells and free cells in the bone/implant interface are also noteworthy. Therefore, another tentative role we present here is this exosome-dependent control of lineage commitment that is mediated by RhoA activity, and specifically *via* its effects on ROCK-mediated cytoskeletal tension. RhoA GTPase and RhoA/ROCK signaling, as a central regulator of contractility in many cells, has been proven to be critical to proliferation and differentiation in numerous studies. RhoA GTPase is even sufficient to mediate the switch in human mesenchymal stem cell (hMSC) commitment between adipogenic and osteogenic fates. The activation of RhoA can facilitate cytoskeleton remodeling (cell spreading), formation of adherens junctions, and downstream integrin signaling, such as TGF-β (Wang et al., 2012), ERK/Runx2, and the Wnt/PCP signaling pathway, which serve notable roles in osteogenic differentiation (Khatiwala et al., 2009; Li et al., 2015). All these imply that the activation of the RhoA/ROCK signaling pathway supports the preferential commitment of the progenitor cells to osteogenic differentiation, which may elucidate the mechanism of exosome treatment for new bone formation from a novel perspective.

Despite the interesting attraction between exosomes and cells and the obvious changes in the behavior of MSCs on Ti surfaces, these results are limited to the conventional single-cell model, especially considering that implant osseointegration is a complex process that involves osteogenesis, osteoinduction, osteoconduction, and remodeling of bone (Qin et al., 2016; Li et al., 2017). This complicated process involves multiple cells such as stem cells, osteoclasts/osteoblasts, and monocytes/macrophages (Sima and Glogauer, 2003). As the recruitment of monocytes/macrophages is crucial in the early stage of osseointegration, studies on the function of exosomes in monocyte–macrophage systems would be critical and interesting (Kang et al., 2020). Another issue that merits further study is whether the same exosomes have different roles in various cellular contexts: for example, whether the exosomes derived from BMSCs have different effects on stem cells, osteoblasts, and osteoclasts.

Even though exosome-based modification of implant surfaces is in its infancy, it exhibits promising prospects to enhance bone–implant integration, especially for patients with preexisting systemic illnesses. In order to achieve exosome implant coating and successful assembly on the titanium surface, the coating technique should be investigated and improved. More research is required to determine the optimum exosome concentration for coatings. The initial binding of exosomes might be stabilized by the antibody/ligand binding, like a covalent bond on the chemically modified titanium surfaces *via* intentional biological modification. Patients with systemic diseases such as diabetes and osteoporosis, having a lower implant survival, might benefit from these implants coated with exosomes due to the docking of exosomes to their parent cells, which suggests a more precise approach to treatment (Tsolaki et al., 2009).

**FIGURE 7:**
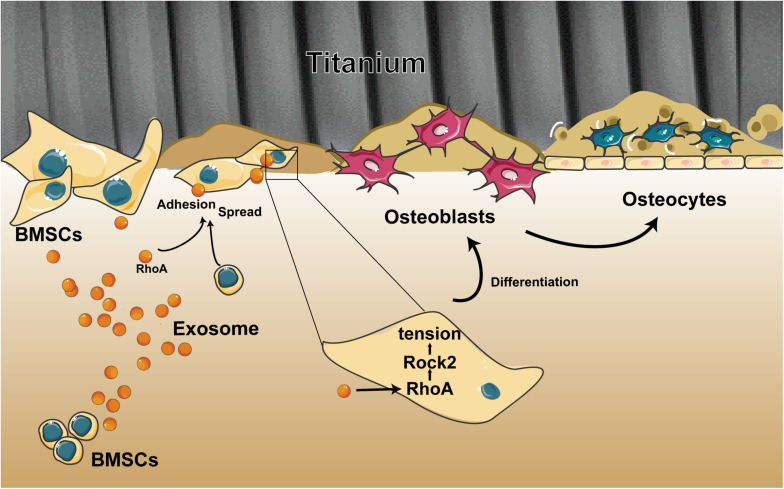
Schematic of the multiple effects of exosomes on rat bone marrow stem cells (MSCs) on a Ti surface. Free/adhered BMSCs release exosomes. These BMSC-exosomes identify homologous cells, aid in efficient cellular internalization, and promote the adhesion, spread, and osteogenesis differentiation of BMSCs on the Ti surface.

Overall, we demonstrated the ability of exosomes to target cells and change their biological behaviors, which is an opportunity that opens up new ways to devise targeted therapies to facilitate the osseointegration of a dental implant. Our research described three important findings (Figure 7): (i) BMSC-exosomes derived by either adherent cells or suspension cells can be internalized efficiently by cells and to change the biological behaviors of cells. (ii) When exosomes are ingested by suspension cells, they advance cell attachment to the Ti surface. (iii) When exosomes are ingested by adherent cells or have been bound to cells as a whole and adhered to a Ti surface, they promote cell spreading and osteogenic differentiation. All these findings present new insights into the regulatory role of exosomes on cells on a Ti surface and provide a novel way to modify the surface of Ti implants.

## Conclusion

To conclude, we report that exosomes exhibit efficient cellular internalization and take part in the regulation of cell morphology as well as biological behaviors. Our results demonstrate the novel insight that exosomes can promote the activity of BMSCs in the process of implant osseointegration and could be used for implant surface modification. Further experiments are required to investigate the functional roles of exosomes derived from diverse cells in a more complex model system to study the dynamics and regulation of multicellular populations.

## Data Availability Statement

The raw data supporting the conclusions of this article will be made available by the authors, without undue reservation.

## Ethics Statement

The animal study was reviewed and approved by the Ethics Committee for Animal Research at Zhejiang University (Ethics approval number: ZJU20200075).

## Author Contributions

ZX and ZC conceived and designed the experiments. YL, QJ, HX, and CY performed the experiments. YL and YY analyzed the data. YL and XZ wrote the first draft of the manuscript. All authors contributed to manuscript revision and read and approved the submitted version.

## Conflict of Interest

The authors declare that the research was conducted in the absence of any commercial or financial relationships that could be construed as a potential conflict of interest.
