# Supplementary material for: Exosomes Enhance Adhesion and Osteogenic Differentiation of Initial Bone Marrow Stem Cells on Titanium Surfaces
Source: Front Cell Dev Biol. 2020 Nov 5;8:583234. doi: 10.3389/fcell.2020.583234 (PMC7674173; doi:10.3389/fcell.2020.583234)
Supplement: Supplementary file 1 [file Table_1.DOCX]

**Supplementary Table 1**

Primers used for real-time PCR

| Gene | Forward primer sequence (5’-3’) | Reverse primer sequence (3’-5’) |
| --- | --- | --- |
| RhoA | GTGCCCACAGTGTTTGAGA | ATCGGTATCTGGGTAGGAG |
| Rock2 | TCGACAGCTTGCTCCAAACA | CGCGCATGTGGTGTATGTAT |
| Osterix | CGGCAAGGTGTACGGCAAGG | GAGCAGAGCAGACAGGTGAACTTC |
| OCN | AGACTCCGGCGCTACCTCAAC | GGCGTCCTGGAAGCCAATGTG |
| ALP | CACGGCGTCCATGAGCAGAAC | CAGGCACAGTGGTCAAGGTTGG |
